# Recent advances in understanding and management of acquired thrombocytopenia

**DOI:** 10.12688/f1000research.12309.1

**Published:** 2018-01-17

**Authors:** Srikanth Nagalla, Ravindra Sarode

**Affiliations:** 1 Division of Hematology/Oncology, UT Southwestern Medical Center, Dallas, TX, USA; 2 Division of Transfusion Medicine and Hemostasis, Department of Pathology, UT Southwestern Medical Center, Dallas, TX, USA

**Keywords:** Thrombocytopenia, immune, heparin, thrombotic thrombocytopenic purpura, atypical hemolytic uremic

## Abstract

There are numerous congenital and acquired causes of thrombocytopenia. Thrombocytopenia could be a result of decreased bone marrow production, increased consumption, increased destruction, splenic sequestration or a combination of these causes. In this review, we have focused on some of the serious acquired causes of thrombocytopenia. There have been some significant advances in our understanding of the pathophysiology, diagnostic testing, and treatment of immune thrombocytopenia, heparin-induced thrombocytopenia, thrombotic thrombocytopenic purpura, and atypical hemolytic uremic syndrome over the past five years. These advances have resulted in a significant decrease in mortality and morbidity of patients with these disorders. Despite these advances, we are still faced with numerous unanswered questions in the pathophysiology and management of these complex thrombocytopenic disorders.

## Introduction

There are numerous causes for thrombocytopenia, defined as a platelet count below 150 × 10 ^9^/L, and they could be broadly classified as congenital and acquired. Acquired thrombocytopenia could be immune or non-immune. Thrombocytopenia could be a result of decreased marrow production, increased destruction or sequestration/consumption in the periphery, or a combination of decreased production and sequestration. Initial steps in the evaluation of thrombocytopenia include review of the peripheral blood smear to exclude pseudo-thrombocytopenia due to platelet clumping. The peripheral blood smear may also provide clues toward other causes of thrombocytopenia when combined with the complete blood count and a good patient history and physical examination. Platelet size and presence of schistocytes, polychromasia, or spherocytes are some of the other features on the peripheral blood smear that help in diagnosing the etiology of thrombocytopenia. Thrombocytopenia could be a harbinger of a serious underlying medical condition such as thrombotic thrombocytopenic purpura (TTP) or heparin-induced thrombocytopenia (HIT). Therefore, it is always important for the treating physician to evaluate thrombocytopenia in a timely fashion so that the treatment for some of the serious conditions is not delayed. The current review will focus primarily on the recent updates in the pathophysiology and management of some of the serious acquired causes of thrombocytopenia encountered in the hospital setting ( Table 1).

**Table 1.** Recent advances in understanding and management of thrombocytopenia.

Disease entity	Recent updates
ITP	1. Absence of platelet-bound antibodies may predict non-response to rituximab. 2. Patients with lower platelet counts and increased platelet reactivity have lower risk of bleeding. 3. Multiple cycles of pulsed dexamethasone may result in higher long-term remission rates. 4. Rituximab combined with high-dose dexamethasone in the first-line setting could result in higher response rates. 5. Patients who are refractory to one of the TPO-RAs may respond to the other. 6. Combining TPO-RA might be an option in refractory ITP. 7. Recombinant thrombopoietin was safe and efficacious in pregnant patients.
HIT	1. Prerequisites for an immunogenic HIT antigen have been better understood. 2. The atomic structure of the PF4-heparin complex has been defined. 3. PF4 released from platelets during a bacterial infection can bind to the bacterial cell wall, resulting in a priming step for HIT. 4. PF4-heparin complexes can activate, complement, and bind to the complement receptor 2 (CD21) on B cells to enhance antibody production. 5. Spontaneous HIT developments in the absence of heparin exposure can occur after orthopedic procedures. 6. Dynamic mechanical thromboprophylaxis may increase the incidence of HIT antibody formation. 7. Low-molecular-weight heparin use instead of heparin results in significant cost savings due to the reduced incidence of HIT. 8. Fondaparinux is safe and effective in the treatment of acute HIT. 9. Accumulating evidence suggests safety and efficacy of direct oral anticoagulants in HIT. 10. Refractory HIT with prolonged thrombocytopenia may benefit from intravenous immunoglobulin.
TTP	1. Hyperbilirubinemia and plasma-free hemoglobin can falsely decrease ADAMTS13 activity in fluorescence resonance energy transfer (FRET) assay. 2. Persistent ADAMTS13 activity of less than 10% within seven days of daily PLEX could be associated with worse outcomes. 3. Upfront use of rituximab reduces exacerbation, refractoriness, number of PLEX sessions, and possibly hospital stay. 4. Bortezomib is very promising in rituximab-refractory/relapsed TTP. 5. Caplacizumab reverses pathophysiology instantly with a potential to reduce early mortality. 6. ADAMTS13 activity of less than 10% alone is not sufficient for TTP relapse and requires another trigger. 7. Cardiac and mesenteric ischemia is common. 8. Delayed neurocognitive defects occur in some patients.
aHUS	1. Only about 60% of the patients have an identifiable genetic mutation. 2. A modified Ham test can distinguish aHUS from TTP. 3. The duration of eculizumab therapy is not clearly defined at the present time.

ADAMTS13, a disintegrin and metalloproteinase with thrombospondin type 1 motif, member 13; aHUS, atypical hemolytic uremic syndrome; HIT, heparin induced thrombocytopenia; ITP, immune thrombocytopenia; PF4, platelet factor 4; PLEX, plasma exchange; TPO-RA, thrombopoietin receptor agonist; TTP, thrombotic thrombocytopenic purpura.

## Immune thrombocytopenia

Most patients presenting with immune thrombocytopenia (ITP) either are asymptomatic or have minor muco-cutaneous bleeding. ITP remains a diagnosis of exclusion because there is no reliable diagnostic test to detect platelet autoantibodies ^1^. However, a rapid response to intravenous immunoglobulin (IVIG) or steroids, in addition to being therapeutic, might also aid in the diagnosis of ITP. Platelet autoantibodies or T cell-mediated autoimmunity or both play a role in the pathophysiology of ITP resulting in platelet destruction and reduced platelet production ^2^. Although platelet autoantibodies are not used in the diagnosis of ITP, recent work suggests that the lack of platelet-bound antibodies might predict non-responsiveness to rituximab therapy in patients with ITP ^3^. The bleeding phenotype varies significantly in patients with similar platelet counts and may be related to factors such as concomitant medications, age of the patient, and platelet reactivity. There is some evidence to suggest that ITP patients with lower platelet counts and increased platelet reactivity have a lower risk of bleeding ^4^. Intracranial hemorrhage is the most dreaded complication of ITP, and history of significant bleeding from other sites predicts for intracranial hemorrhage ^5^.

Steroids are the mainstay in the first-line treatment of ITP. A two- to four-week course of prednisone (1 mg/kg and taper) or four days of pulsed high-dose (40 mg daily) dexamethasone given every two to four weeks are the two commonly used steroid regimens ^1^. High-dose dexamethasone was found in one study to be more effective in inducing long-term remission compared with prednisone ^6^. Multiple cycles of pulsed dexamethasone may result in higher long-term remission rates compared with prednisone or a single cycle of dexamethasone ^6, 7^. Two other retrospective studies showed conflicting results when comparing the efficacy of dexamethasone with that of prednisone ^8, 9^. Further prospective studies are needed to conclusively prove the efficacy of one steroid over the other. Given the convenience of a short-course, high-dose dexamethasone regimen over a longer duration of prednisone, we recommend high-dose dexamethasone over prednisone for the initial treatment of ITP ^10^. Treating physicians should be aware of the fact that a sustained response with high-dose dexamethasone might require multiple cycles of therapy, although some proportion of the patients might not achieve remission despite this approach. IVIG administered at a dose of 1 g/kg for one or two days is the other first-line treatment option ^1^. Given the faster platelet response to IVIG compared with steroids, IVIG is used in conjunction with steroids in patients with severe thrombocytopenia requiring urgent procedures or presenting with moderate to severe bleeding.

Before the advent of thrombopoietin receptor agonists (TPO-RAs), splenectomy and rituximab were the commonly used second-line agents. A recent single-center cohort study compared 83 splenectomized ITP patients with 83 non-splenectomized ITP patients. Splenectomy resulted in an overall response rate of 52% at 192 months ^11^. There was an increase in the venous thromboembolic events and serious infections resulting in hospitalization in the splenectomized population without a decrease in the overall survival. A recent population-matched cohort study demonstrated that splenectomized patients had a 50% overall increased five-year stroke risk compared with the disease-matched cohort (absolute risk 3% versus 2.3%) but that there was no increase in the five-year stroke risk in ITP patients undergoing splenectomy ^12^. Another recent large single-center study confirmed the curative nature of splenectomy in more than 50% of the patients undergoing the procedure, and corticosteroid dependence predicted for sustained response after splenectomy ^13^. Taking into consideration the high cure rates and the opportunity to avoid long-term medications, we recommend considering splenectomy as the initial second-line treatment option in young steroid-dependent ITP patients.

Rituximab, an anti-CD20 monoclonal antibody, can result in 50 to 60% initial response rates, although only about 20% of the patients maintain the response at five years ^14^. A recent randomized control trial comparing rituximab with placebo demonstrated no difference in the complete remission rates between the two arms at 18 months ^15^. Rituximab, when combined with one to three cycles of high-dose dexamethasone in a first-line setting, could result in higher response rates than dexamethasone alone ^16– 18^.

Two TPO-RAs, romiplostim and eltrombopag, were initially used in the third-line setting but have rapidly moved up the ladder to second-line status. These agents have transformed the management for ITP. Long-term follow-up of these agents has demonstrated that they are highly effective and safe in the treatment of patients with ITP ^19– 21^. TPO-RA could also be used in the first-line setting, in conjunction with steroids and IVIG, to treat severe thrombocytopenia and significant bleeding. There is small proportion of patients who are refractory to one of the agents but may respond to the other ^22, 23^. There is also some experience in combining the two agents, given their different target sites on the thrombopoietin receptor, in patients refractory to single-agent, second-line therapy ^23^. TPO-RA could also be used in combination with other immunosuppressive therapies to improve the response rates in refractory ITP patients ^24^.

Although ITP in pregnancy can generally be treated with steroids and IVIG, there is an occasional need for other effective therapies when the patients do not respond to the first-line agents. A recent study showed that recombinant thrombopoietin was safe and efficacious in increasing the platelet count in 31 pregnant women with ITP ^25^. Unlike the previously studied pegylated human recombinant megakaryocytic growth and development factor (PEG-rHuMGDF), which resulted in the formation of anti-thrombopoietin antibodies ^26, 27^, the new recombinant and glycosylated form of thrombopoietin was similar to endogenous thrombopoietin, and anti-thrombopoietin antibodies were not detected in the limited sample size of patients. These pregnant females with ITP were refractory to the first-line therapy of steroids or IVIG (or both) with some signs and symptoms of bleeding and platelet counts of less than 30 × 10 ^9^/L. About 75% of patients responded with platelet counts increasing to 30–100 × 10 ^9^/L or more than 100 × 10 ^9^/L. TPO-RA and recombinant thrombopoietin might have an important role in the management of pregnant refractory ITP patients.

## Heparin-induced thrombocytopenia

Despite the widespread use of direct oral anticoagulants (DOACs) and other parenteral anticoagulants, unfractionated heparin (UFH) still plays an important role in the management of hospitalized patients. Hence, understanding the pathophysiology and management of HIT is still very relevant to the treating physician ^28^. HIT is a disease of multiple paradoxes. It is a disease in which an anticoagulant causes thrombosis and thrombocytopenia is associated with thrombosis rather than bleeding. In HIT, unlike in other thrombocytopenias, platelet transfusions increase the risk of clotting. Simply stopping the offending agent (heparin) is not adequate treatment unlike other drug-induced thrombocytopenias and vitamin K antagonists anticoagulant is contraindicated in the treatment of acute HIT.

HIT antibodies are induced by platelet factor 4 (PF4)-heparin or endogenous glycosaminoglycan complexes. A subset of these IgG antibodies can cross-link Fcγ receptor IIA (FcRγIIA), causing platelet and monocyte activation that results in consumptive thrombocytopenia and venous and/or arterial thrombosis ^29^. There are multiple prerequisites for an immunogenic HIT antigen. The immunogenicity depends on distinct stoichiometric ratios of PF4-heparin complexes, and the complexes reach a size of more than 670 kDa by charge neutralization. Immunogenic polyanions with anti-parallel beta-sheet content of more than 30% and an energy release of more than 4,000 calories per molPF4 for effective antibody binding are the other prerequisites ^30, 31^. The atomic structure of the PF4-heparin complex was worked out in 2015, although the synthetic pentasaccharide, fondaparinux, was used in the complex instead of heparin ^32^. PF4 released from platelets during a bacterial infection can bind to the bacterial cell wall, resulting in a priming step that could result in the formation of antibodies against PF4-heparin complex when exposed to heparin at a later time ^33^. PF4-heparin complexes can activate complement and bind to the complement receptor 2 (CD21) on B cells, enhancing the antibody production ^34^. A better understanding of the HIT pathophysiology would lead to the development of better diagnostic tests and effective anticoagulants to treat the condition.

Spontaneous HIT develops in the absence of exposure to any heparin and is common in patients undergoing orthopedic procedures ^35^. The tissue damage as a result of total hip or knee arthroplasty can lead to platelet activation, polyanion release, and formation of PF4-polyanion complexes. Dynamic mechanical thromboprophylaxis might add to the tissue damage, platelet activation, and polyanion release, increasing the incidence of HIT antibody formation ^36^. In the setting of total knee arthroplasty, the incidence of HIT antibody formation was similar in patients receiving dynamic mechanical thromboprophylaxis with or without heparin/fondaparinux. Since they were identified by enzyme-linked immunosorbent assay (ELISA), it is not clear whether these antibodies are platelet-activating.

Reducing the incidence of HIT with the use of low-molecular-weight heparins (LMWHs) instead of UFH results in significant cost savings with an ‘avoid heparin’ protocol ^37^. Although the pharmacy-and-therapeutics committee of many hospitals prefers UFH over LMWH for venous thromboembolism prophylaxis because of the cost differential between the two drugs, a single case of complicated HIT with thrombosis can wipe out all the upfront cost savings.

Fondaparinux is safe and effective in the treatment of acute HIT ^38, 39^. The time taken to achieve therapeutic levels, less risk of bleeding, and shorter hospital stay resulting in cost savings are some of the advantages of fondaparinux over argatroban. We recommend fondaparinux over parenteral direct thrombin inhibitors in the treatment of acute HIT in patients who are not critically ill and have good renal function. There is accumulating evidence for the safety and efficacy of DOACs in the treatment of HIT ^40, 41^. These medications, like fondaparinux, would definitely help with reducing the length of hospital stay and patient convenience. Until there are more data, we do not recommend DOACs in the initial treatment of acute HIT, but they could be used in patients after platelet recovery.

A small proportion of patients with HIT might have refractory disease with prolonged thrombocytopenia despite therapeutic anticoagulation. IVIG could be used in conjunction with anticoagulation to help with platelet recovery ^42^.

## Thrombotic thrombocytopenic purpura

Microangiopathic hemolytic anemia (MAHA) and thrombocytopenia are a common denominator in multiple disease states. Of these, TTP is the easiest to diagnose since it is caused by a severe ADAMTS13 (a disintegrin and metalloproteinase with a thrombospondin type 1 motif, member 13 or von Willebrand cleaving protease) deficiency (<10%) ^43^. TTP could be acquired due to autoantibodies to ADAMTS13 or congenital due to severe deficiency of ADAMTS13. We recommend testing for ADAMTS13 in every patient with MAHA and thrombocytopenia when TTP is part of the differential diagnosis, although treatment of TTP should not be delayed while awaiting the results ^44^. Sepsis and cirrhosis can decrease ADAMTS13 activity but generally not less than 10% ^43^. Hyperbilirubinemia (>10 mg/dL) and free plasma hemoglobin, however, could give falsely deficient levels (<10%) due to interference in the fluorescence resonance energy transfer (FRET) assay ^43, 45^. IgG antibodies against ADAMTS13 are detected in 75–80% of the patients by the functional Bethesda method in acquired TTP; however, remaining patients may have non-neutralizing antibodies often detected by an ELISA ^46^. Because ADAMTS13 assay is often sent to a reference laboratory, most patients with unexplained MAHA and thrombocytopenia are started on plasma exchange (PLEX). If the ADMTS13 is more than 10%, PLEX should be discontinued and other causes of MAHA and thrombocytopenia should be investigated for appropriate treatment such as metastatic malignancy, malignant hypertension, and atypical hemolytic uremic syndrome (aHUS) ^44^. The blood for ADAMTS13 testing should preferably be drawn prior to instituting PLEX, although levels are often low in acquired TTP even after a couple of days of PLEX ^47^. Not achieving ADAMTS13 activity levels of more than 10% within seven days of instituting daily PLEX could be associated with worse outcomes ^47^.

PLEX should be started as soon as the clinical diagnosis of TTP is made. The currently available plasma preparations are all equivalent in terms of their outcomes in TTP ^48^. Glucocorticoids are often started simultaneously. Once the diagnosis is confirmed to be autoimmune TTP, rituximab is routinely used in the treatment of relapsed or refractory TTP patients. However, there is evidence to support upfront use of rituximab in acute TTP to reduce exacerbation, refractoriness, number of PLEX sessions, and possibly hospital stay ^46^. Although this approach might result in overtreatment of half of the patients with TTP, the decreased morbidity and mortality in patients with refractory or relapsed TTP would outweigh the overtreatment issue. Most physicians use rituximab 375 mg/m ^2^ weekly for four doses, although our personal experience with a lower dose of rituximab at 100 mg weekly for four doses appears promising. There is a clinical trial under way to study the efficacy of low-dose rituximab (ClinicalTrials.gov Identifier: NCT01554514). Bortezomib is very promising as an adjunct to PLEX in the treatment of refractory or relapsed acquired TTP or both ^49, 50^. We recommend using bortezomib in refractory and/or relapsed TTP patients who do not respond to rituximab. Daily subcutaneous caplacizumab, a nanobody against von Willebrand factor (VWF) A-1 domain preventing its interaction with GPIb on platelets, resulted in faster platelet responses in acute TTP compared with a placebo when used along with PLEX ^51^. This nanobody is not a cure; however, by preventing the interaction between VWF and platelets, it causes rapid reversal of the underlying pathogenetic mechanism, resulting in a potential to reduce early mortality. The drug is being studied further in a phase 3 trial.

Some patients achieving clinical remission may have ADAMTS13 activity of less than 10%. Nonetheless, ADAMTS13 activity of less than 10% alone is not sufficient for TTP relapse and requires a trigger such as infection, pregnancy, or concomitant inflammatory conditions increasing VWF levels ^52^. Although measuring ADAMTS13 activity during remission and preemptively treating with rituximab for moderately to severely low ADAMTS13 activity is a commonly used approach at some institutions, this practice needs further evaluation. We recommend following these patients carefully because of the higher rate of TTP recurrence in this population. Since this is a very episodic disease, education of patients for signs and symptoms of relapse is a crucial part of the management plan. In addition to the neurological and renal manifestations, TTP could result in cardiac and mesenteric ischemia ^53, 54^. Patients could commonly have elevated cardiac troponin without any clinical cardiac abnormalities ^53^. Similarly, long-term neurocognitive defects are recognized in patients with TTP ^55, 56^.

## Atypical hemolytic uremic syndrome

aHUS results from a dysregulated complement system, leading to cell and tissue damage ^57^. Although MAHA, thrombocytopenia, and oliguric renal failure are the three common presenting features, aHUS is a systemic disease that can involve any organ system. aHUS, unlike Shiga toxin-producing *Escherichia coli* HUS, does not present with the prodrome of bloody diarrhea and often is recurrent. Mutations in the genes encoding for complement proteins, complement regulatory factors, or autoantibodies to complement factors, when combined with another stressor such as infection or surgery, often result in the development of aHUS. Only about 60% of patients with aHUS have an identifiable genetic mutation ^58^. Mutations in the gene encoding for factor H are the most common among patients with aHUS. The other defects involve mutations in CD46, factor I, *C3*, factor B, thrombomodulin, diacylglycerol kinase ε, and CFHR1/3 deficiency with anti-factor H autoantibodies. Since mutation testing and screening for antibodies to complement proteins are not available readily, diagnosis of aHUS is usually made on the basis of clinical features and an ADAMTS13 activity of more than 10%. A modified Ham test has shown some promise in distinguishing aHUS and TTP serum samples ^59^. Genetic mutations or the autoantibodies in aHUS result in the activation of alternate pathway (AP); when *PIGA* null TF-1 cells (PNH-like cells) are added to the serum samples, aHUS serum samples result in a positive test (cell death) because of the activated AP compared with the TTP samples.

If TTP is in the differential diagnosis, PLEX is started until ADAMTS13 activity results. But if the presenting features are classic for aHUS or if the ADAMTS13 activity is more than 10%, the appropriate management would be to start eculizumab, an anti-C5 antibody, immediately. Eculizumab is effective in improving renal function and platelet count and halting tissue damage from the AP activation ^60^. The optimal duration of eculizumab therapy in aHUS is not clearly defined at the present time ^61^.

## Conclusions

In summary, acute thrombocytopenia is a common clinical presenting feature in many serious hematological conditions. There have been some significant advances in our understanding of the pathophysiology, diagnostic testing, and treatment of these disorders over the past five years ( Table 1). Unfortunately, ITP remains a diagnosis of exclusion; however, there are many newer treatment options available for ITP, drastically improving the outcome of patients with ITP. Similarly, we have a better understanding of HIT pathophysiology, and this is paving the way for development of better diagnostic and therapeutic options for HIT, including IVIG. Availability of drugs such as caplacizumab would help in preventing early deaths due to rapid reversal of pathophysiology of TTP. B lymphocyte- and plasma cell-directed therapies have improved the response rates in refractory TTP and decreased the relapses. Finally, the use of eculizumab in complement-mediated aHUS has significantly decreased morbidity and mortality.

## References

[1] Neunert C Lim W Crowther M: The American Society of Hematology 2011 evidence-based practice guideline for immune thrombocytopenia. *Blood.* 2011;117(16):4190–207. 10.1182/blood-2010-08-302984 21325604

[2] Cines DB Cuker A Semple JW: Pathogenesis of immune thrombocytopenia. *Presse Med.* 2014;43(4 Pt 2):e49–59. 10.1016/j.lpm.2014.01.010 24630266

[3] Porcelijn L Huiskes E Schipperus M: Lack of detectable platelet autoantibodies is correlated with nonresponsiveness to rituximab treatment in ITP patients. *Blood.* 2017;129(25):3389–91. 10.1182/blood-2016-11-751719 28468795

[4] Middelburg RA Carbaat-Ham JC Hesam H: Platelet function in adult ITP patients can be either increased or decreased, compared to healthy controls, and is associated with bleeding risk. *Hematology.* 2016;21(9):549–51. 10.1080/10245332.2016.1180097 27159138

[5] Melboucy-Belkhir S Khellaf M Augier A: Risk factors associated with intracranial hemorrhage in adults with immune thrombocytopenia: A study of 27 cases. *Am J Hematol.* 2016;91(12):E499–E501. 10.1002/ajh.24529 27528011

[6] Matschke J Müller-Beissenhirtz H Novotny J: A Randomized Trial of Daily Prednisone versus Pulsed Dexamethasone in Treatment-Naïve Adult Patients with Immune Thrombocytopenia: EIS 2002 Study. *Acta Haematol.* 2016;136(2):101–7. 10.1159/000445420 27189086

[7] Wei Y Ji XB Wang YW: High-dose dexamethasone vs prednisone for treatment of adult immune thrombocytopenia: a prospective multicenter randomized trial. *Blood.* 2016;127(3):296–302; quiz 370. 10.1182/blood-2015-07-659656 26480931

[8] Nakazaki K Hosoi M Hangaishi A: Comparison between pulsed high-dose dexamethasone and daily corticosteroid therapy for adult primary immune thrombocytopenia: a retrospective study. *Intern Med.* 2012;51(8):859–63. 10.2169/internalmedicine.51.7005 22504239

[9] Sakamoto K Nakasone H Tsurumi S: Prednisone versus high-dose dexamethasone for untreated primary immune thrombocytopenia. A retrospective study of the Japan Hematology & Oncology Clinical Study Group. *J Thromb Thrombolysis.* 2014;37(3):279–86. 10.1007/s11239-013-0939-3 23686644

[10] Mithoowani S Gregory-Miller K Goy J: High-dose dexamethasone compared with prednisone for previously untreated primary immune thrombocytopenia: a systematic review and meta-analysis. *Lancet Haematol.* 2016;3(10):e489–e496. 10.1016/S2352-3026(16)30109-0 27658982

[11] Thai LH Mahévas M Roudot-Thoraval F: Long-term complications of splenectomy in adult immune thrombocytopenia. *Medicine (Baltimore).* 2016;95(48):e5098. 10.1097/MD.0000000000005098 27902585 PMC5134764

[12] Rørholt M Ghanima W Farkas DK: Risk of cardiovascular events and pulmonary hypertension following splenectomy - a Danish population-based cohort study from 1996–2012. *Haematologica.* 2017;102(8):1333–41. 10.3324/haematol.2016.157008 28572164 PMC5541868

[13] Guan Y Wang S Xue F: Long-term results of splenectomy in adult chronic immune thrombocytopenia. *Eur J Haematol.* 2017;98(3):235–41. 10.1111/ejh.12821 27753191

[14] Chugh S Darvish-Kazem S Lim W: Rituximab plus standard of care for treatment of primary immune thrombocytopenia: a systematic review and meta-analysis. *Lancet Haematol.* 2015;2(2):e75–81. 10.1016/S2352-3026(15)00003-4 26687612

[15] Ghanima W Khelif A Waage A: Rituximab as second-line treatment for adult immune thrombocytopenia (the RITP trial): a multicentre, randomised, double-blind, placebo-controlled trial. *Lancet.* 2015;385(9978):1653–61. 10.1016/S0140-6736(14)61495-1 25662413

[16] Gudbrandsdottir S Birgens HS Frederiksen H: Rituximab and dexamethasone vs dexamethasone monotherapy in newly diagnosed patients with primary immune thrombocytopenia. *Blood.* 2013;121(11):1976–81. 10.1182/blood-2012-09-455691 23293082

[17] Zaja F Baccarani M Mazza P: Dexamethasone plus rituximab yields higher sustained response rates than dexamethasone monotherapy in adults with primary immune thrombocytopenia. *Blood.* 2010;115(14):2755–62. 10.1182/blood-2009-07-229815 20130241

[18] Chapin J Lee CS Zhang H: Gender and duration of disease differentiate responses to rituximab-dexamethasone therapy in adults with immune thrombocytopenia. *Am J Hematol.* 2016;91(9):907–11. 10.1002/ajh.24434 27220625

[19] Cines DB Gernsheimer T Wasser J: Integrated analysis of long-term safety in patients with chronic immune thrombocytopaenia (ITP) treated with the thrombopoietin (TPO) receptor agonist romiplostim. *Int J Hematol.* 2015;102(3):259–70. 10.1007/s12185-015-1837-6 26201709

[20] Elgebaly AS Ashal GE Elfil M: Tolerability and Efficacy of Eltrombopag in Chronic Immune Thrombocytopenia: Meta-Analysis of Randomized Controlled Trials. *Clin Appl Thromb Hemost.* 2017;23(8):928–37. 10.1177/1076029616663849 27572890

[21] Wong RSM Saleh MN Khelif A: Safety and efficacy of long-term treatment of chronic/persistent ITP with eltrombopag: final results of the EXTEND study. *Blood.* 2017;130(23):2527–2536. 10.1182/blood-2017-04-748707 29042367

[22] Khellaf M Viallard JF Hamidou M: A retrospective pilot evaluation of switching thrombopoietic receptor-agonists in immune thrombocytopenia. *Haematologica.* 2013;98(6):881–7. 10.3324/haematol.2012.074633 23445876 PMC3669443

[23] Kuter DJ Macahilig C Grotzinger KM: Treatment patterns and clinical outcomes in patients with chronic immune thrombocytopenia (ITP) switched to eltrombopag or romiplostim. *Int J Hematol.* 2015;101(3):255–63. 10.1007/s12185-014-1731-7 25586660

[24] Mahévas M Gerfaud-Valentin M Moulis G: Characteristics, outcome, and response to therapy of multirefractory chronic immune thrombocytopenia. *Blood.* 2016;128(12):1625–30. 10.1182/blood-2016-03-704734 27354722

[25] Kong Z Qin P Xiao S: A novel recombinant human thrombopoietin therapy for the management of immune thrombocytopenia in pregnancy. *Blood.* 2017;130(9):1097–1103. 10.1182/blood-2017-01-761262 28630121

[26] Basser RL O'Flaherty E Green M: Development of pancytopenia with neutralizing antibodies to thrombopoietin after multicycle chemotherapy supported by megakaryocyte growth and development factor. *Blood.* 2002;99(7):2599–602. 10.1182/blood.V99.7.2599 11895799

[27] Li J Yang C Xia Y: Thrombocytopenia caused by the development of antibodies to thrombopoietin. *Blood.* 2001;98(12):3241–8. 10.1182/blood.V98.12.3241 11719360

[28] Onwuemene O Arepally GM: Heparin-induced thrombocytopenia: research and clinical updates. *Hematology Am Soc Hematol Educ Program.* 2016;2016(1):262–8. 10.1182/asheducation-2016.1.262 27913490 PMC6142447

[29] Tutwiler V Madeeva D Ahn HS: Platelet transactivation by monocytes promotes thrombosis in heparin-induced thrombocytopenia. *Blood.* 2016;127(4):464–72. 10.1182/blood-2013-11-539262 26518435 PMC4731847

[30] Brandt S Krauel K Gottschalk KE: Characterisation of the conformational changes in platelet factor 4 induced by polyanions: towards in vitro prediction of antigenicity. *Thromb Haemost.* 2014;112(1):53–64. 10.1160/TH13-08-0634 24671506

[31] Kreimann M Brandt S Krauel K: Binding of anti-platelet factor 4/heparin antibodies depends on the thermodynamics of conformational changes in platelet factor 4. *Blood.* 2014;124(15):2442–9. 10.1182/blood-2014-03-559518 25150299

[32] Cai Z Yarovoi SV Zhu Z: Atomic description of the immune complex involved in heparin-induced thrombocytopenia. *Nat Commun.* 2015;6:8277. 10.1038/ncomms9277 26391892 PMC4580983

[33] Krauel K Pötschke C Weber C: Platelet factor 4 binds to bacteria, [corrected] inducing antibodies cross-reacting with the major antigen in heparin-induced thrombocytopenia. *Blood.* 2011;117(4):1370–8. 10.1182/blood-2010-08-301424 20959601

[34] Khandelwal S Lee GM Hester CG: The antigenic complex in HIT binds to B cells via complement and complement receptor 2 (CD21). *Blood.* 2016;128(14):1789–99. 10.1182/blood-2016-04-709634 27412887 PMC5054694

[35] Warkentin TE Basciano PA Knopman J: Spontaneous heparin-induced thrombocytopenia syndrome: 2 new cases and a proposal for defining this disorder. *Blood.* 2014;123(23):3651–4. 10.1182/blood-2014-01-549741 24677540

[36] Bito S Miyata S Migita K: Mechanical prophylaxis is a heparin-independent risk for anti-platelet factor 4/heparin antibody formation after orthopedic surgery. *Blood.* 2016;127(8):1036–43. 10.1182/blood-2015-06-651620 26659923 PMC4768427

[37] McGowan KE Makari J Diamantouros A: Reducing the hospital burden of heparin-induced thrombocytopenia: impact of an avoid-heparin program. *Blood.* 2016;127(16):1954–9. 10.1182/blood-2015-07-660001 26817956

[38] Kang M Alahmadi M Sawh S: Fondaparinux for the treatment of suspected heparin-induced thrombocytopenia: a propensity score-matched study. *Blood.* 2015;125(6):924–9. 10.1182/blood-2014-09-599498 25515959

[39] Warkentin TE: How I diagnose and manage HIT. *Hematology Am Soc Hematol Educ Program.* 2011;2011(1):143–9. 10.1182/asheducation-2011.1.143 22160026

[40] Davis KA Davis DO: Direct acting oral anticoagulants for the treatment of suspected heparin-induced thrombocytopenia. *Eur J Haematol.* 2017;99(4):332–5. 10.1111/ejh.12921 28672052

[41] Warkentin TE Pai M Linkins LA: Direct oral anticoagulants for treatment of HIT: update of Hamilton experience and literature review. *Blood.* 2017;130(9):1104–13. 10.1182/blood-2017-04-778993 28646118

[42] Padmanabhan A Jones CG Pechauer SM: IVIg for Treatment of Severe Refractory Heparin-Induced Thrombocytopenia. *Chest.* 2017;152(3):478–85. 10.1016/j.chest.2017.03.050 28427966 PMC5812774

[43] Sadler JE: What's new in the diagnosis and pathophysiology of thrombotic thrombocytopenic purpura. *Hematology Am Soc Hematol Educ Program.* 2015;2015(1):631–6. 10.1182/asheducation-2015.1.631 26637781 PMC4777280

[44] Shah N Rutherford C Matevosyan K: Role of ADAMTS13 in the management of thrombotic microangiopathies including thrombotic thrombocytopenic purpura (TTP). *Br J Haematol.* 2013;163(4):514–9. 10.1111/bjh.12569 24111495

[45] Meyer SC Sulzer I Lämmle B: Hyperbilirubinemia interferes with ADAMTS-13 activity measurement by FRETS-VWF73 assay: diagnostic relevance in patients suffering from acute thrombotic microangiopathies. *J Thromb Haemost.* 2007;5(4):866–7. 10.1111/j.1538-7836.2007.02438.x 17408415

[46] Joly BS Coppo P Veyradier A: Thrombotic thrombocytopenic purpura. *Blood.* 2017;129(21):2836–46. 10.1182/blood-2016-10-709857 28416507

[47] Wu N Liu J Yang S: Diagnostic and prognostic values of ADAMTS13 activity measured during daily plasma exchange therapy in patients with acquired thrombotic thrombocytopenic purpura. *Transfusion.* 2015;55(1):18–24. 10.1111/trf.12762 24953079

[48] Toussaint-Hacquard M Coppo P Soudant M: Type of plasma preparation used for plasma exchange and clinical outcome of adult patients with acquired idiopathic thrombotic thrombocytopenic purpura: a French retrospective multicenter cohort study. *Transfusion.* 2015;55(10):2445–51. 10.1111/trf.13229 26173755

[49] Eskazan AE: Bortezomib therapy in patients with relapsed/refractory acquired thrombotic thrombocytopenic purpura. *Ann Hematol.* 2016;95(11):1751–6. 10.1007/s00277-016-2804-x 27590601

[50] Patriquin CJ Thomas MR Dutt T: Bortezomib in the treatment of refractory thrombotic thrombocytopenic purpura. *Br J Haematol.* 2016;173(5):779–85. 10.1111/bjh.13993 27009919

[51] Peyvandi F Scully M Kremer Hovinga JA: Caplacizumab for Acquired Thrombotic Thrombocytopenic Purpura. *N Engl J Med.* 2016;374(6):511–22. 10.1056/NEJMoa1505533 26863353

[52] Page EE Kremer Hovinga JA Terrell DR: Clinical importance of ADAMTS13 activity during remission in patients with acquired thrombotic thrombocytopenic purpura. *Blood.* 2016;128(17):2175–8. 10.1182/blood-2016-06-724161 27625362 PMC5084608

[53] Benhamou Y Boelle PY Baudin B: Cardiac troponin-I on diagnosis predicts early death and refractoriness in acquired thrombotic thrombocytopenic purpura. Experience of the French Thrombotic Microangiopathies Reference Center. *J Thromb Haemost.* 2015;13(2):293–302. 10.1111/jth.12790 25403270

[54] Mariotte E Azoulay E Galicier L: Epidemiology and pathophysiology of adulthood-onset thrombotic microangiopathy with severe ADAMTS13 deficiency (thrombotic thrombocytopenic purpura): a cross-sectional analysis of the French national registry for thrombotic microangiopathy. *Lancet Haematol.* 2016;3(5):e237–45. 10.1016/S2352-3026(16)30018-7 27132698

[55] Falter T Schmitt V Herold S: Depression and cognitive deficits as long-term consequences of thrombotic thrombocytopenic purpura. *Transfusion.* 2017;57(5):1152–62. 10.1111/trf.14060 28337761

[56] Han B Page EE Stewart LM: Depression and cognitive impairment following recovery from thrombotic thrombocytopenic purpura. *Am J Hematol.* 2015;90(8):709–14. 10.1002/ajh.24060 25975932 PMC4509840

[57] Jokiranta TS: HUS and atypical HUS. *Blood.* 2017;129(21):2847–56. 10.1182/blood-2016-11-709865 28416508 PMC5445567

[58] Rodríguez de Córdoba S Hidalgo MS Pinto S: Genetics of atypical hemolytic uremic syndrome (aHUS). *Semin Thromb Hemost.* 2014;40(4):422–30. 10.1055/s-0034-1375296 24799305

[59] Gavriilaki E Yuan X Ye Z: Modified Ham test for atypical hemolytic uremic syndrome. *Blood.* 2015;125(23):3637–46. 10.1182/blood-2015-02-629683 25862562 PMC4784297

[60] Legendre CM Licht C Muus P: Terminal complement inhibitor eculizumab in atypical hemolytic-uremic syndrome. *N Engl J Med.* 2013;368(23):2169–81. 10.1056/NEJMoa1208981 23738544

[61] Rodriguez E Barrios C Soler MJ: Should eculizumab be discontinued in patients with atypical hemolytic uremic syndrome? *Clin Kidney J.* 2017;10(3):320–2. 10.1093/ckj/sfx024 28616209 PMC5466109

